# Association between sex and body mass index as mediated by temperament in a nonclinical adult sample

**DOI:** 10.1007/s40519-018-0617-8

**Published:** 2018-11-17

**Authors:** Włodzimierz Oniszczenko, Ewa Stanisławiak

**Affiliations:** 1 Faculty of Psychology, University of Warsaw, Stawki 5-7, 00-183 Warsaw, Poland; 2 Faculty of Psychology, University of Finance and Management, Pawia 55, 01-030 Warsaw, Poland

**Keywords:** BMI, Sex, Temperament, Adults

## Abstract

**Purpose:**

The main purpose of the present study was to investigate the association between sex and body mass index (BMI) as mediated by the temperament traits postulated by the regulative theory of temperament.

**Methods:**

A group of 317 subjects, including 122 men and 195 women 18–82 years old (*M* = 31.93; SD = 12.64 years), was recruited from the general population to participate in the study. A cross-sectional design was applied in this study. Temperament was assessed using the formal characteristics of behaviour–temperament inventory revised version (FCB–TIR). In the sample, BMIs ranged from 16.51 to 35.56 kg/m^2^ (*M* = 23.31 kg/m^2^; SD = 3.61 kg/m^2^).

**Results:**

The male group had significantly higher BMI, briskness and endurance levels and significantly lower perseveration, sensory sensitivity and emotional reactivity levels compared to the female group. No significant differences between these groups were noted in terms of activity and rhythmicity. The correlations between BMI and briskness and between BMI and endurance were positive, while the correlations between BMI and perseveration and between BMI and activity were negative. The correlations among BMI, sensory sensitivity, emotional reactivity and rhythmicity were not statistically significant. The mediation analysis showed that briskness and endurance were significant partial mediators of the relationship between sex and BMI.

**Conclusions:**

Sex and two temperament traits, briskness and endurance, were the best predictors of BMI. Briskness and endurance partially mediated the relationship between sex and BMI.

**Level of evidence:**

Descriptive cross-sectional study. Level V.

## Introduction

Body mass index (BMI) is an easy-to-perform screening for weight category and is commonly used to classify overweight and obesity in adults. BMI is also used as an indicator of the risk of developing obesity [1]. Risk factors for the development of obesity include genetics, metabolism, behaviour and environmental and socioeconomic factors. In addition, several authors have described the role of human personality in the development of obesity.

Some studies have also shown that men have higher BMI levels compared to women, although this pattern may depend on the culture and age of the subjects. For example, Kuan, Ho, Shuhaili, Siti and Gudum [2] examined a Malaysian sample of students and found that more males than females were overweight, while more females were underweight. Furthermore, this study demonstrated that females preferred an ideal figure that was underweight, whereas males chose an overweight figure as their ideal model. Similar results were obtained in a study of Indian students [3]. The highest BMI level in men relative to women was observed in a large Western European sample [4] as well as in a Central European sample [5]. In contrast, Kodjebacheva, Kruger, Rybarczyk and Cupal [6] found that African American women had a higher BMI than African American men. Meanwhile, Furthner et al. [7] demonstrated a lack of gender differences in BMI in the youngest age group (aged 10–14) of their study, yet BMI during adolescence (at the age of 17) was higher in boys and significantly decreased in girls. A recent analysis showed similar gender trends of BMI increasing over a 40-year period in adults living in 200 different countries [8].

In addition, several authors have described gender differences in the relationship between personality and BMI. Brummett et al. [9] demonstrated that neuroticism was significantly and positively related to BMI in females. Extraversion was also positively related to BMI in males, yet this latter relation was non-significant in females. Meanwhile, a significantly negative correlation between conscientiousness and BMI was found for both males and females. According to Dietrich et al. [10], Behavioural Inhibition Scale (BIS) and Behavioural Approach Scale (BAS) responses were associated with BMI in a gender-specific manner; a negative relationship was observed for men and a positive relationship for women. Emery and Levine [11] demonstrated in their meta-analysis that impulsivity was positively associated with BMI. In an earlier review, Gerlach, Herpertz and Loeber [12] emphasised that neuroticism, impulsivity and sensitivity to reward are risk factors for a high BMI, while conscientiousness and self-control may have a protective function in relation to BMI increase.

The present study was based on the regulative theory of temperament (RTT) formulated by Strelau [13]. According to this theory, traits are manifested in the energetic characteristics (response intensity) and temporal characteristics (speed, tempo and mobility) of behaviour. The RTT assumes that temperamental traits are present in humans from infancy and implies that these traits are important components of human behaviour across all life situations. Additionally, temperament regulates the relationship between a subject and his/her environment and appears, generally in difficult and extreme situations, to determine individuals’ possibilities of external stimulation processing and preferred methods of regulation. Seven temperament traits are postulated as follows: briskness (BR; the tendency towards quick reactions and maintenance of a high tempo while performing activities), perseveration (PE; the tendency to continue and/or repeat a behaviour after the cessation of stimuli that evoked the behaviour), sensory sensitivity (SS; the ability to react to sensory stimuli of low stimulative value and to detect minor differences between sensory stimuli values), emotional reactivity (ER; the tendency to react intensely to emotogenic stimuli, manifested in high emotional sensitivity and low emotional resistance), endurance (EN; the ability to adequately react to situations demanding long-lasting and highly stimulative activity), activity (AC; the tendency to undertake highly stimulative behaviours or to supply external stimulation through undertaken behaviour) and rhythmicity (RT; the regularity of the time intervals between homogenous reactions, which manifests in eating and sleeping habits and lifestyle drive).

RTT traits can play a similar role in disorders and better-known personality traits. ER and PE are positively correlated with anxiety [13], neuroticism [14] and harm avoidance [15]. BR, EN and AC are positively correlated with extraversion [14] and novelty-seeking [15]. Oniszczenko, Stanisławiak, Dembińska-Krajewska and Rybakowski [16] showed that ER and PE are positively correlated with anxious, cyclothymic, depressive and irritable affective temperaments, while the hyperthymic temperament is positively correlated with BR, SS, EN and AC. In our previous study involving bariatric patients [17], we suggested that low BR, EN and AC levels may serve as risk factors for the development of obesity. Low levels of these traits with accompanying high levels of PE and ER may be potential risk factors for affective disorders in obese patients, especially in women.

Several studies have additionally suggested that sex may modify the relationship between personality and BMI. Faith et al. [18] demonstrated that, among women, increases in BMI were significantly associated with increased neuroticism and reduced extraversion; among men, BMI increases were associated with increased extraversion and psychoticism. Further, Brummett et al. [9] showed that neuroticism is negatively correlated with BMI in females, while extraversion is positively correlated with BMI in males. The latter authors also found a negative correlation between conscientiousness and BMI in males and a positive correlation in females, and the magnitude of this correlation was stronger in females. Jokela et al. [19] suggest in their meta-analysis that high conscientiousness, which reflects high self-control, orderliness and adherence to social norms, was associated with lower obesity risk both in men and women. Dietrich et al. [10] showed that the relationship between the behavioural inhibition system (sensitivity to punishment) and the behavioural activation system (sensitivity to reward) was negatively correlated with BMI in men and positively correlated with BMI in women. In another study, Blanch and Aluja [20] demonstrated low positive correlations between BMI and neuroticism and between BMI and sociability in males along with low negative correlations between BMI and impulsive sensation seeking and between BMI and activity in females. Hintsanen et al. [21] showed that higher novelty-seeking predicted higher BMI in men and women, whereas lower reward dependence predicted higher BMI in women.

The purpose of this study was to investigate the association between sex and BMI as mediated by the temperament traits postulated by the RTT in a nonclinical adult sample. We hypothesised that sex may directly and indirectly affect BMI through the mediation of temperament traits (BR, EN and AC).

## Methods

### Participants

The sample was comprised of 317 subjects recruited from the general population, including 122 men and 195 women 18–82 years old (*M* = 31.93; SD = 12.64). Men and women were equivalent in terms of age [*t* (315) = − 1.10, *p* = 0.274]. Height ranged from 150 to 198 cm (*M* = 171.88 cm; SD = 9.79 cm), and their weight ranged from 40 to 110 kg (*M* = 69.27 kg; SD = 14.28 kg). BMI values fell between 16.51 and 35.56 kg/m^2^ (*M* = 23.31 kg/m^2^, SD = 3.61 kg/m^2^).

With respect to participant characteristics, 175 participants (55.7%) had post-secondary education, 132 participants (42%) had secondary education and 7 participants (2.2%) had primary education (3 participants did not provide information about their education). Two hundred and three participants (65.5%) were unmarried, 93 (30%) were married and 14 (4.5%) were divorced (7 participants did not provide information about their marital status). In regard to location, 36 participants (11.4%) lived in rural areas, 49 (15.5%) in small towns and 232 (73.2%) in large cities.

This study was part of a larger research project investigating personality. Participants were directly recruited by interviewers, who contacted students of various universities as well as their colleagues, friends, parents and, in the case of working students, their co-workers.

The inclusion criterion was age ≥ 18 years old. The exclusion criteria were any self-reported physical (somatic) or mental problems. The study was anonymous, and participation was voluntary. Participants were not remunerated.

### Measures

Temperament traits were assessed using Cyniak-Cieciura, Zawadzki and Strelau’s [22] Formal Characteristics of Behaviour–Temperament Inventory, Revised Version (FCB–TIR). This inventory consists of 100 items divided into 15 items per subscale, with the exception of 10 items for the RT subscale. Participants answered each item using a 4-point Likert scale ranging from strongly disagree (1) to strongly agree (4). The Cronbach’s alphas of the current sample were as follows: BR (*α* = 0.81), PE (*α* = 0.79), SS (*α* = 0.80), ER (*α* = 0.87), EN (*α* = 0.86), AC (*α* = 0.86) and RT (*α* = 0.86). Higher total scores for the FCB–TIR subscales indicated higher levels of the respective traits.

### Statistical analysis

**Fig. 1:**
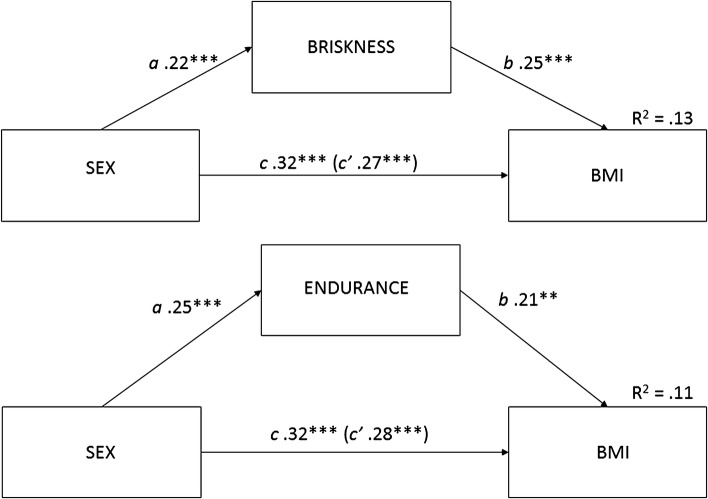
Relationship between sex and BMI as mediated by the temperament traits of briskness (upper) and endurance (lower). The R2 value for the sex-briskness model and for the sex-endurance model was placed above the BMI symbol. **p < 0.01; ***p < 0.001. Path coefficients are standardized regression weights; *c* = the direct effect of sex on BMI without the influence of briskness/endurance, *a, b* = the indirect effect of sex on BMI through briskness/endurance, *c*′ = the direct effect of sex on BMI including the influence of briskness/endurance

Statistical analyses were performed using IBM SPSS Statistics for Windows v. 24 [23]. Data normality was checked based on the skewness and kurtosis values following the application of criteria specified by Tabachnick and Fidell [24]. Student’s t-test was employed for two independent samples to identify differences between the male and female groups concerning age, BMI and temperament traits. Effect sizes of the t-tests were estimated using Cohen’s d, since effect sizes generally provide more interpretable and quantitative descriptions of the size of an effect. According to Cohen [25], d values of 0.8, 0.5 and 0.2 represent large, medium and small effect sizes, respectively. The relationships among the variables were examined using partial correlation coefficients controlled for sex effect. A multivariate linear regression analysis was also used to estimate the effects of sex and temperament traits as BMI predictors. The mediation analyses were conducted following the Baron and Kenny [26] approach. According to this model, in the first step, a simple regression analysis was conducted, with sex as an independent variable predicting BMI as a dependent variable (path c in Fig. 1). In the second step, a simple regression analysis with sex predicting the mediator (path a in Fig. 1) was conducted. In the third step, a simple regression analysis with the mediator predicting BMI (path b in Fig. 1) was conducted. In the fourth step, a multiple regression analysis with sex and the mediator predicting BMI (path c′ in Fig. 1) was conducted. The significance of the indirect effects was confirmed using Sobel’s test [27].

## Results

The skewness and kurtosis analyses showed that all variables were normally distributed. Skewness varied from − 0.31 (briskness) to 0.58 (BMI), while kurtosis ranged from − 0.31 (rhythmicity) to 0.03 (sensory sensitivity).

Table 1 provides descriptive statistics for the male and female subgroups and the differences in the studied variables between subgroups. The male group had significantly higher BMI, briskness and endurance values (Cohen’s d values were 0.69, 0.46 and 0.52, respectively, suggesting a medium effect) and significantly lower sensory sensitivity, perseveration and emotional reactivity values compared to the female group. (Cohen’s d values were 0.27, 0.63 and 0.52, respectively, suggesting a small, medium and large effect, respectively.) However, no significant differences between these groups were noted on the activity and rhythmicity scales.

**Table 1:** Mean values and standard deviations of BMI and temperament traits in males (*n* = 122) and females (*n*  = 195)

Variables	Males, *M* (SD)	Females, *M* (SD)	*t* test, (*df* = 315)	Cohen’s *d*
Age	32.91 (12.26)	31.31 (12.87)	− 1.09	0.13
BMI (kg/m^2^)	24.75 (3.11)	22.41 (3.62)	− 5.91***	0.69
Briskness	46.07 (6.11)	43.10 (6.64)	− 4.00***	0.46
Perseveration	40.93 (5.56)	44.63 (6.14)	5.41***	0.63
Sensory sensitivity	44.13 (6.13)	45.79 (6.03)	2.37*	0.27
Emotional reactivity	35.36 (7.42)	41.31 (7.23)	7.06***	0.81
Endurance	37.87 (8.06)	33.88 (7.24)	− 4.56***	0.52
Activity	37.43 (7.80)	37.88 (7.69)	0.50	0.06
Rhythmicity	21.71 (5.84)	21.64 (5.83)	− 0.11	0.01

*M* mean, *SD* standard deviation, *df* degrees of freedom

*p < 0.05; **p < 0.01; ***p < 0.001

Table 2 presents the partial correlation coefficients between age, FCB–TIR subscales and BMI. Regarding the relationships between BMI and the seven FCB–TIR subscales, the partial correlations while controlling for sex were small. The correlations between BMI and briskness/endurance were positive (*r* = 0.19, *p* < 0.001 and *r* = 0.14, *p* < 0.05, respectively), while the correlations between BMI and perseveration/activity were negative (*r* = − 0.12, *p* < 0.05 and *r* = − 0.15, *p* < 0.001, respectively). Additionally, the correlations of BMI with sensory sensitivity, emotional reactivity and rhythmicity were not statistically significant. Age was positively correlated with BMI (*r* = 0.43, *p* < 0.001), briskness (*r* = 0.17, *p* < 0.01) and rhythmicity (*r* = 0.28, *p* < 0.001) and negatively correlated with perseveration (*r* = − 0.24, *p* < 0.001) and activity (*r* = − 0.22, *p* < 0.001), whereas its correlations with sensory sensitivity, emotional reactivity and endurance were not statistically significant.

**Table 2:** Partial correlations between age, FCB–TIR scales and BMI while controlling for sex across the entire sample (*n* = 317)

Variables	Age	BMI	BR	PE	SS	ER	EN	AC	RT
Age		0.43***	0.17**	− 0.24***	0.04	− 0.03	0.05	− 0.22***	0.28***
BMI			0.19***	− 0.12*	0.05	− 0.03	0.14*	− 0.15**	0.03
Briskness (BR)				− 0.08	0.26***	− 0.21***	0.31***	0.16**	0.09
Perseveration (PE)					0.10	0.47**	− 0.24***	0.03	− 0.03
Sensory sensitivity (SS)						− 0.01	0.06	0.21***	0.06
Emotional reactivity (ER)							− 0.37***	− 0.16**	0.01
Endurance (EN)								0.21***	− 0.17**
Activity (AC)									− 0.07

*BMI* body mass index, *BR* briskness, *PE* perseveration, *SS* sensory sensitivity, *ER* emotional reactivity, *EN* endurance, *AC* activity, *RT* rhythmicity

*p < 0.05; **p < 0.01; ***p < 0.001

The multivariate linear regression analysis indicated that the best BMI predictors were sex (*ß* = 0.24, *p* < 0.001), briskness (*ß* = 0.17, *p* < 0.01), endurance (*ß* = 0.13, *p* < 0.05) and activity (*ß* = − 0.19, *p* < 0.001) [*R*^2^ adjusted = 0.17, *F*(8, 308) = 8.93, *p* < 0.001]. Sex accounted for 5%, briskness for 2%, endurance for 1% and activity for 3% of BMI variance. Since sex did not differentiate activity, it was excluded from the mediation analysis.

The mediation model was used twice, separately for briskness and endurance, to explore whether sex affected BMI directly or did so indirectly through the traits of briskness and endurance in our adult sample. The results of both analyses are shown in Fig. 1. Both briskness (Sobel test values: *Z* = 2.65, *p* < 0.01) and endurance (Sobel test values: *Z* = 2.28, *p* < 0.05) were significant partial mediators of the relationship between sex and BMI.

## Discussion

The current study aimed to understand the relationships between sex and BMI as mediated by the temperament traits the RTT postulated. We assumed that the differences in the RTT traits between men and women play an important role in determining BMI. Additionally, we hypothesised that through the mediation of temperament traits (briskness, endurance and activity), sex may directly and indirectly affect BMI. The results showed that sex may directly affect BMI, though with only a medium effect size [25]. Sex may also affect BMI indirectly, with briskness and endurance as mediators, although in our study these effects were small.

Our results are in line with worldwide trends indicating that men have a higher overall BMI compared to women [8]. In the current sample, men had higher briskness and endurance levels, which suggest a tendency towards higher levels of physical activity. Higher briskness implies a stronger tendency towards quick reaction, maintaining a high tempo of activity and shifting easily in response to changing environmental conditions. In turn, endurance as a temperament trait determines one’s ability to handle distractors as well as fatigue [13]. Although the briskness manifests itself mainly in human physical activity, its level does not translate into a low BMI. Conversely, we showed that briskness was positively correlated with the BMI level (see Table 2). We assumed that the observed result was associated with more general personality dimensions, such as extraversion. Briskness, endurance and activity are positively correlated, both phenotypically and genetically, with extraversion in the five-factor personality theory [14]. Higher briskness and endurance levels in men compared to women suggest that men have a more extraverted behaviour style than women. Activity and gregariousness are typical of extraversion. Activity can mean increased energy expenditure, but gregariousness can be associated with excessive eating and drinking, which are important social rituals. As Sutin [28] explained, when eating in a group, individuals tend to consume more food and drink than when eating by themselves, and extraversion is associated with eating more in response to external (e.g. social) cues. It is probable that men are more likely to engage in ‘extraverted’ lifestyles that contribute to excess weight (e.g. a ‘more energetic’ diet conducive to obesity) or that male diets are characterized by the overconsumption of unhealthy foods suggested as potential aetiological factors in relation to obesity [29]. Another aspect of extraversion is that extraverts may be frequent mobile phone users because they are social, talkative and gregarious in nature [30]. We believe that the use of mobile phones may reduce the need for physical activity, yet this behaviour may be somewhat problematic. As Lepp et al. [31] showed, cell phone or smartphone use, like many other traditional sedentary behaviours, may disrupt physical activity and may be partially responsible for the rise in obesity, e.g. by facilitating access to fast food. In general, the positive correlations of BMI with briskness and endurance—two traits strongly associated with extraversion—may support the hypothesis that predicted more of a positive relationship between extraversion and BMI for men than for women [9, 29].

In the present study, we did not find any activity-related gender differences, even though this trait is not directly related to physical or motor activity but rather with the tendency to undertake highly stimulating behaviours that can lead to prolonged emotional distress. Individuals with a higher level of activity are more at risk for psychological distress than individuals with low activity levels. Therefore, activity may indirectly contribute to BMI regulation through emotional distress accompanying highly stimulating behaviour. As Bourdier et al. [32] demonstrated, psychological distress is positively correlated with the BMI of men and women, with emotional eating and food addiction serving as mediators between psychological distress and BMI. In the present study, we showed that activity was negatively correlated with BMI. In other words, individuals who engage in highly stimulating behaviour or behaviours that involve a stimulating environmental input seem to be at a lower risk for obesity. We suspect that this may be due to greater exposure to distress and higher emotional costs, which reduce energy resources. As Claassen et al. [33] expressed in their review, higher stress while simultaneously controlling for depressive symptoms may be related to lower BMI and obesity.

In our study, women scored higher than men on perseveration and emotional reactivity (i.e. indicative of higher neuroticism); only perseveration weakly and negatively correlated with BMI. In our sample, neuroticism did not seem to play as prominent role as the BMI risk factor. In an earlier study, we suggested that both emotional reactivity and perseveration may serve as risk factors in the development of mood disorders in obese people [17].

The present study did not find rhythmicity to play a role in BMI level, although other studies have suggested a relationship between daily rhythmicity in locomotor activity and BMI [34].

Significant positive correlations between age and BMI as well as between age and briskness and rhythmicity were found in the analysis, though negative correlations between age and BMI and activity were present. Individuals’ increased BMI may be due to a decrease in physical activity and a tendency to assume a sedentary lifestyle as they age [35] or to lifestyle changes involving energy expenditure and dietary intake [36]. According to Wadden et al. [37], a higher BMI level at an older age may be explained not only by the low level of physical activity but also by high-fat and high-sugar food intake, genetic and/or endocrine factors and some medications. As we showed (see Table 2), the increase in BMI is accompanied by the decline in activity during one’s lifespan. A decline in activity and an increase in rhythmicity with age suggest that older people are less involved in stimulating behaviours and may prefer serenity and an orderly life rather than a stressful environment. A decline in activity may also suggest the decrease of another personality trait, such as openness to experience, which is positively correlated with activity [14]. As Mõttus et al. [38] indicated, the dietary habits of older people may be less related to how controlled they are and more related to their levels of openness and emotional and social adjustment. A low level or lack of intellectual curiosity (i.e. openness) could be a potential barrier to a healthy diet and may consequently contribute to an increase in BMI. In turn, the briskness that increases with age can be explained by the adaptive function of this temperamental trait in everyday life. As Rizzuto et al. [39] showed, extroverted people (higher in briskness), who are characterized by high self-efficacy, are prone to healthier behaviours and better health, which may result in longer survival.

In summary, our nonclinical study illustrated the role of temperament traits in the mutual relationship between sex and BMI. Briskness and endurance (i.e. indicative of extraversion) were two temperament traits positively correlated with BMI in adults of both sexes in that high levels of these traits were associated with high BMI. Males who had higher levels of BR and EN compared to women also had a greater risk of overweight/obesity development than women. Taking into account the relatively young age of the participants in our current study, we assume that high levels of BR and EN may contribute to an increased risk of developing overweight/obesity, mainly as a result of the extraverted lifestyle and diet of people at this age [3, 33]. It is worth noting that women face social pressures to pay attention to their appearance and maintain a slim figure, which likely reduces the risk of overweight/obesity [2].

While the high level of briskness and endurance, especially in men, may contribute to the increase in BMI level, the development of overweight/obesity is conducive to decreasing both traits. The development of overweight/obesity can decrease levels of BR and EN because obese people are often characterized by reduced physical activity, slowness and low mobility behaviour as well as low endurance and a low fatigue threshold [17].

Interpretation of our results must consider several potential limitations of our study. First, the cross-sectional design of the study prevented us from assessing the temporal relationship between the variables and thus from drawing any causal interpretations. Longitudinal data are needed to test for possible reciprocal relationships between personality and BMI. Second, we had no information from participants in regard to lifestyle, diet, regularity of meal intervals throughout the day, snacking between meals, physical activity, work activity or medical treatment, all of which may also serve as mediators between sex and BMI. In particular, no control was applied for metabolic diseases, which could affect the results of this study. Third, we relied on self-reported height and weight, which are vulnerable to reporting biases. All of these limitations should be addressed in future research.

Despite these limitations, our research provided new insight into the role of temperament in the aetiology of obesity in adults of both sexes. Temperament traits may play a significant role in obesity as risk factors. This knowledge can help in the development of prevention measures for people at risk of overweight/obesity.
